# Flexible Composites with Rare-Earth Element Doped Polycrystalline Particles for Piezoelectric Nanogenerators

**DOI:** 10.3390/mi15111280

**Published:** 2024-10-22

**Authors:** Yanzhe Fan, Zihan Jia, Zhuo Zhang, Shengfei Gu, Wenya Du, Dabin Lin

**Affiliations:** 1 School of Opto-Electronical Engineering, Xi’an Technological University, Xi’an 710021, China; yanzhefan2024@163.com (Y.F.); zhuozhang2021@163.com (Z.Z.); 2 Young Talent Program from China Association for Science and Technology and Ministry of Education, Beijing Science Center, Beijing 100190, China; zihanjia@bjsc.net.cn; 3 School of Information and Communication Engineering, Dalian University of Technology, Dalian 116024, China; 2642208063@mail.dlut.edu.cn; 4 Media Lab, Massachusetts Institute of Technology, Cambridge, MA 02139, USA

**Keywords:** piezoelectric nanogenerators, energy harvesting, self-powered devices, polymer-matrix composites

## Abstract

Energy harvesting plays an important role in advancing personalized wearables by enabling continuous monitoring, enhancing wearable functionality and facilitating sustainable solutions. We aimed to develop a flexible piezoelectric energy harvesting system based on inorganic piezoelectric materials that convert mechanical energy into electricity to power a wide range of mobile and portable electronic devices. There is significant interest in flexible piezoelectric energy harvesting systems that use inorganic piezoelectric materials due to their exceptional physical features and prospective applications. Herein, we successfully demonstrated a flexible piezoelectric nanogenerator (PENG) designed by the co-doped rare-earth element ceramics (RE-PMN-PT) embedded in PVDF and PDMS composite film and attained a significant output performance while avoiding electrical poling process. The impact of dielectric characteristics on the electrical output of nanogenerators was investigated, together with the structure of the composites. The Sm/La-PMN-PT particles effectively amplify both the voltage and current output, showcasing their potential to power portable and wearable devices, as demonstrated by their capacity to illuminate LEDs. The maximal output power of 2 mW was correlated with the high voltage (220 V) and current (90 µA) of Sm/La-PMN-PT/PVDF, which demonstrated that the device has the potential for energy harvesting in biomedical applications.

## 1. Introduction

The field of wearable and portable electronics has experienced significant and swift expansion in recent years [1]. These technologies are widely utilized in many applications, including wireless sensor systems, smart skin for health monitoring, human–machine interfaces, security systems, and others [2]. The integration of these electronic devices into real-life situations has imposed further requirements on the power supply [3,4]. Traditional chemical batteries are characterized by inflexible construction, significant weight, and electrolyte solutions that have detrimental effects on the environment. There is an increasing interest in energy harvesting devices designed for nano/microsystems that can power themselves [5,6].

Many efforts have been made to investigate new energy conversion systems, such as nanogenerators, that can capture energy from the surrounding environment to power wearable and portable electronics [7]. Triboelectric nanogenerator (TENG) and piezoelectric nanogenerator (PENG) are two effective methods for producing electricity from surrounding mechanical vibrations [8,9], such as those caused by human body motion, vehicle motions, water flow, air flow, sound waves, and other mechanical resources. Both of these options provide numerous benefits, such as a versatile design, lightweight construction, eco-friendliness, affordability, and the capability to power low-energy electronic devices by converting biomechanical energy into electricity [10]. Both nanogenerators have demonstrated great output electrical performance, long-term circle stability, mechanical flexibility, and robustness [11]. PENG usually has a faster response speed and a wider frequency response range, higher chemical stability, and mechanical strength and can directly carry out energy conversion without friction processes, which reduces the possibility of material wear and energy loss. Many piezoelectric materials can maintain good performance over a wide temperature range, which makes PENG work effectively in high or low-temperature environments. Piezoelectric materials can be made into thin films or coatings that are easily integrated into a variety of surfaces or structures for a variety of applications.

In recent years, a variety of approaches for improving their performances mainly focus on new material introduction, structure innovation, and micro-surface coupling modification. It has been demonstrated that all these approaches can enhance the output of electrical performance because of their superior piezoelectric properties, enhanced electric charge-discharge network, and improved homogeneous microstructures [12]. An effective method for enhancing the output power of these piezoelectric nanodevices is to incorporate new piezoelectric fillers that possess a higher piezoelectric coefficient [13]. Recently, piezoelectric nanocomposites have attracted much attention, which are produced by dispersing piezoelectric nanoparticles in a flexible matrix, including barium titanate (BaTiO_3_) [13,14,15], lead zirconium titanate (PbZrTiO_3_) [16], (Na, K)NbO_3_, BiNaTiO_3_ [17], Zinc Oxide (ZnO) [18,19]. Compared with other piezoelectric materials, a solid solution (1−x)Pb(Mg_1/3_Nb_2/3_)O_3_−xPbTiO_3_ (PMN-PT) has been widely recognized as a relaxor ferroelectric compound due to the significantly elevated dielectric anomaly caused by the uneven distribution of B-site cations within the perovskite lattice of Pb(Mg_1/3_Nb_2/3_)O_3_ [20]. Generally, PMN-PT ceramics exhibited 2–3 times enhancement of piezoelectric coefficient [21], which resulted in the improvement of the output performance. For example, Li and colleagues conducted a study on a PENG employing 0.7PMN-0.3PT nanorods. The fabricated PENG was able to reach a maximum output voltage of 10.3 V and an output current of 46 nA. These values were 13 times higher for the output voltage and 4.5 times higher for the output current compared to the pure PVDF piezoelectric polymer [22]. The enhancement can be attributed to the direct external stress and shear stress distributed on the nanorods during the mechanical deformation. Different from previous work using low-dimensional piezoceramic fillers, Cheng’s group not only used novel materials (Sm-PMN-PT) but also proposed a new interconnected three-dimensional (3D) skeleton, which showed the effective stress transfer under the external mechanical stimulation, resulting in a greatly enhanced energy harvesting output [23]. The device demonstrated a peak instantaneous power density of around 11.5 µWcm^−2^, which is approximately 16 times greater than that of the typical nanoparticle-based composite. Sm-PMN-PT ceramics were also adopted in Gu’s work, in which a new design of PENG was reported with a 3D intercalation electrode and the utilization of Sm-PMN-PT to conquer low output current density. Due to the increased surface polarization charges caused by boundary interfaces, a maximum peak short-circuit current density of 290 μAcm^−2^ can be obtained, which is 1.61 times the record values of PENG [24]. All these works indicated that the piezoelectric properties of the filler have a strong impact on the output performance. Very recently, co-doped PMN-PT ceramics have been investigated, exhibiting improved piezoelectric performance and thermal stability [25,26]. Nevertheless, to the best of our knowledge, there have been few reports of investigations on the PENG with co-doped PMN-PT ceramics.

In order to obtain the high-voltage and current output characteristics for a flexible device, we designed flexible PENG devices using the rare-earth elements doped polycrystalline particles (Sm/La-PMN-PT) embedded in two different matrix-PVDF and PDMS. The impact of dielectric characteristics on the electrical output of nanogenerators was investigated, along with the structure of the composites. The RE-PMN-PT particles are utilized to increase the output voltage and current, showcasing their capability to power wearable/portable devices by illuminating the LEDs. This study also examined the impact of external load resistance, pushing force, and frequency on the electrical output performance of PENGs, as well as their mechanical stability and durability.

## 2. Materials and Methods

### 2.1. Materials

The PDMS (Sylgard^®^ 184 silicone elastomer base and silicone elastomer curing agent) was purchased from the Dow Corning Corporation, Auburn, MI, USA. Polyvinylidene fluoride (PVDF Solef 1008) powders were purchased from Arkema Chemical Co., Ltd. Chateauroux, France. Indium tin oxide coated PET (surface resistively 60 Ω/sq, 1 ft × 1 ft × 5 mil) was purchased from Aldrich. The Nb_2_O_5_ (99.99%), MgCO_3_ (99%), TiO_2_ (99%), La_2_O_3_ (99.9%), Sm_2_O_3_ (99.9%), and Pb_3_O_4_ (99%) were used as raw materials and employed by Alpha Aesar, Shanghai, China. The NaCl and KCl were purchased from Sinopharm Chemical Reagent Co., Ltd. Shanghai, China.

### 2.2. Synthesis of Sm/La-PMN-PT Polycrystalline

0.5%Sm/2%La-Pb [(Mg_1/3_Nb_2/3_)_0.8_Ti_0.2_]O_3_ (Sm/La-PMN-PT) polycrystalline were prepared using a B site cation precursor method. The MgNb_2_O_6_ precursor materials were first fabricated at 1200 °C for 4 h using Nb_2_O_5_ and MgCO_3_ powders. The Pb_3_O_4_, MgNb_2_O_6_, and TiO_2_ powders were homogeneously combined using zirconium ball milling in the presence of alcohol as a solvent for a duration of 6 h. Subsequently, the blended powders underwent a calcination process at a temperature of 850 °C for a duration of 2 h. Following this, they were subjected to vibratory milling in an alcohol medium along with a binder for a total duration of 6 h. Following a 10 h drying period at a temperature of 80 °C, the powders were compressed into pellets with a thickness of 1 mm and a diameter of 10 mm using a uniaxial pressure of 30 MPa. The binder was incinerated at a temperature of 600 °C for a duration of 2 h, and the samples were then subjected to sintering in the presence of air. This sintering process took place in sealed corundum crucibles at a temperature of 1150 °C for a duration of 6 h.

### 2.3. Characterization of Sm/La-PMN-PT Polycrystalline

The X-ray diffraction (XRD) data generated using the Bruker D8 instrument (Bremen, Germany) were used to determine the crystal structure of the samples. The samples were analyzed for their morphology and elemental composition using a field emission scanning electron microscope (Zeiss Gemini SEM 500, Jena, Germany). The measurements included the generation of EDX elemental maps. In order to conduct more precise electrical measurements, gold electrodes were deposited onto the surface of the samples via a process called sputtering. The specimens were subjected to polarization in silicone oil at a temperature of 25 °C for a duration of 10 min, utilizing a direct current electric field with an intensity of 20 kV/cm. The dielectric characteristics were measured using an LCR meter model E4980AL (Santa Clara, CA, USA).

### 2.4. Design and Fabrication of Composite Film

**Figure 1:**
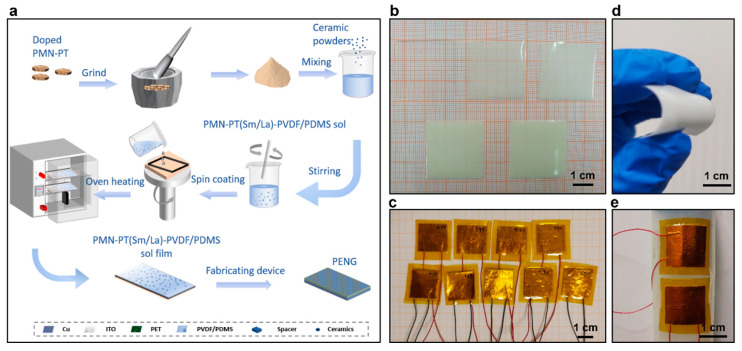
(**a**) Schematic of preparation of Sm/La-PMN-PT based PVDF and PDMS nanocomposite for PENG devices: grinding ceramic disks into powders, mixing powders with PVDF or PDMS solutions, spin coating the mixture, casting the solutions, curing the mixture at high temperature to form the sol film, and fabricating the PENG devices. (**b**) The photo of Sm/La-PMN-PT ceramic disks. (**c**) The photo of Sm/La-PMN-PT/PVDF and Sm/La-PMN-PT/PDMS PENG devices. (**d**) The bending status of the composite film. (**e**) The devices can be wrapped on the surface of the cylinder.

The manufacturing procedure of Sm/La-PMN-PT-PDMS and Sm/La-PMN-PT-PVDF PENG devices is illustrated in Figure 1a. Initially, the ceramic pellets underwent grinding to transform them into a fine powder. Subsequently, the powder was combined with PDMS solutions of different concentrations, specifically 10, 20, 30, and 40 wt.%. The solution was carefully mixed, and any bubbles were removed using degassing. Subsequently, the mixture was applied to the central region of the substrate. The sample was then subjected to spin coating at a speed of 800 rpm for a duration of 30 s to achieve a homogeneous PDMS layer. Following this, the film was cured at a temperature of 80 °C for a period of 2 h, as depicted in Figure 1b. The fabrication process follows the same processes as the manufacture of PVDF films but with different concentrations of 5, 10, 15, and 20 wt.%. The copper tape was affixed to both sides of the thin sheet, as depicted in Figure 1c. Before and after the device fabrication, both composites and devices maintain sufficient flexibility, as shown in Figure 1d,e.

### 2.5. Characterization of the PENG

A universal testing equipment (Instron-5882, Norwood, MA, USA) was employed to determine the stress-strain curve and fracture strength. The Precision LCR Meter (E4980AL, Santa Clara, CA, USA) was employed to determine the dielectric permittivity and loss. Data collection was conducted using a mixed domain oscillometer (MDO3022, Beaverton, OR, USA), an electrometer (KEITHLEY 6517A, Beaverton, OR, USA), and a laptop to measure the open current voltage (VOC) and short-circuit current (ISC) of all devices.

## 3. Results

**Figure 2:**
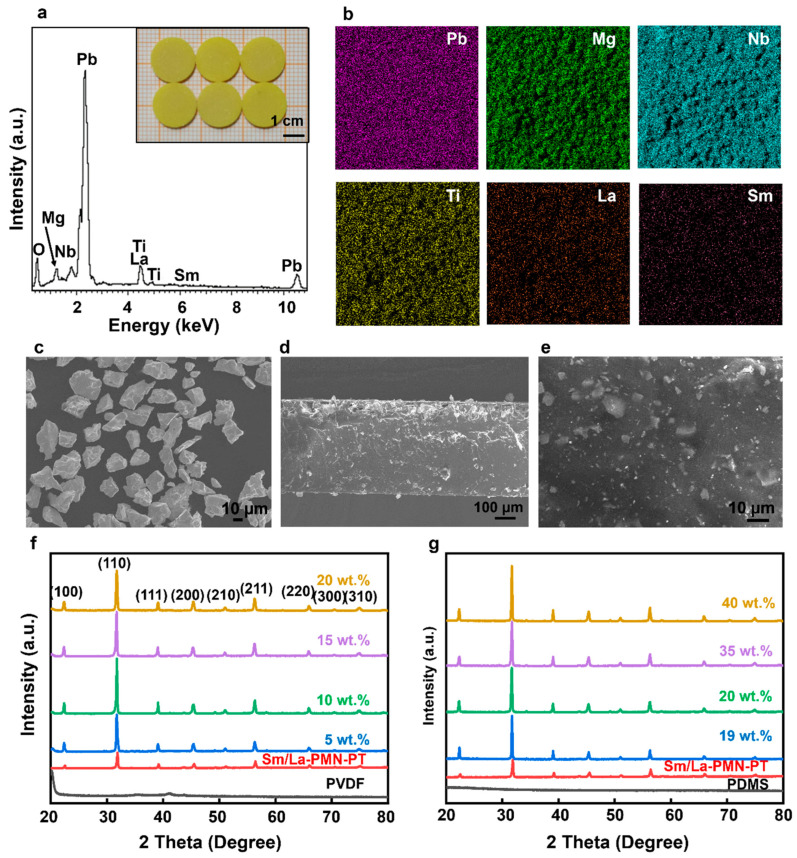
(**a**) The EDX spectra of Sm/La-PMN-PT ceramics in the region. The inset photo shows Sm/La-PMN-PT ceramic disks. (**b**) Different element mappings of Pb, Mg, Nb, Ti, La, and Sm. Morphologies from SEM images: (**c**) the Sm/La-PMN-PT powders, (**d**) the cross-section and (**e**) the surface of Sm/La-PMN-PT/PDMS composite film with 40 wt.% of BT nanoparticles. The X-ray diffraction spectra of various composite films: (**f**) Sm/La-PMN-PT/PVDF and (**g**) Sm/La-PMN-PT/PDMS.

The final Sm/La-PMN-PT ceramic disks are shown in Figure 2a. The EDX spectra in Figure 2a,b confirms the uniform distribution of all elements in the Sm/La-PMN-PT samples. The homogeneous microstructure determines the outstanding piezoelectric properties, including *d*_33_ = 600 pC/N, *ε*_r_ = 10,200, tan*δ* = 0.02, *k*_33_ = 0.68, which are higher than the values of commercial BaTiO_3_, PZT and PMN-PT ceramics [27]. Figure 2c displays highly enlarged SEM pictures of the morphologies of Sm/La-PMN-PT particles. The irregular form of BT nanoparticles is a result of the high-speed ball milling process, and their average size ranges from 10 to 30 µm. Figure 2d,e displays SEM images of the cross-section and surface of the composite film containing 40 wt.% of Sm/La-PMN-PT. These pictures provide extensive information about the morphology of the film, showing that the fillers are uniformly distributed. Figure 2f,g depicts the XRD profile of the PVDF and PDMS composite with varying amounts of crystal fillers, spanning from 20° to 80°. The XRD examination indicated the presence of seven distinct diffraction peaks, namely (100), (110), (111), (200), (210), (211), and (220). These peaks closely match the diffraction pattern expected for the ferroelectric tetragonal phase of PMN-PT (ICSD Collection Code: 161999, PDF#01-076-9083). Furthermore, the presence of diffraction peaks at about 20° and 41°, which correspond to the crystal planes (110)/(200) and (201) of PVDF [15], confirms the existence of the amorphous phase of PDMS.

**Figure 3:**
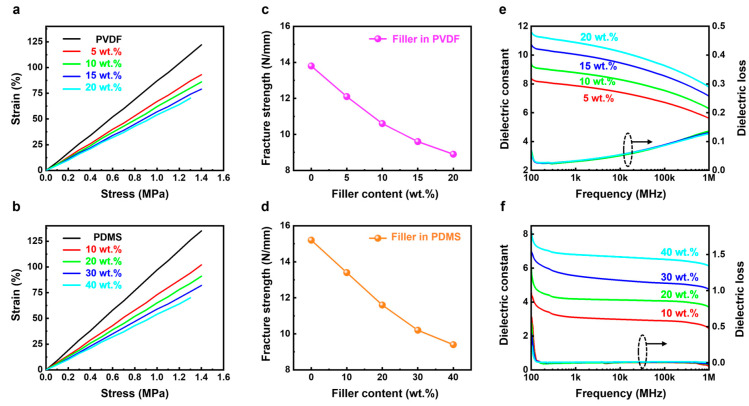
Stress–strain curve of (**a**) Sm/La-PMN-PT/PVDF films and (**b**) Sm/La-PMN-PT/PDMS films with different filler contents. Fracture strength of (**c**) Sm/La-PMN-PT/PVDF films and (**d**) Sm/La-PMN-PT/PDMS films with different filler contents. Dielectric properties of (**e**) Sm/La-PMN-PT/PVDF films and (**f**) Sm/La-PMN-PT/PDMS films with different filler contents.

Mechanical functionality is one of the critical performances for flexible/wearable devices [28,29]. An investigation was conducted on the correlation between strain and stress in both composites, as illustrated in Figure 3a,b. Prior to the addition of the crystal filler, the pure PDMS and PVDF exhibited a reduced strength but an increased elongation at break, as observed. Following the introduction of the filler, there is a reduction in both strength and elongation at break. The obtained results are consistent with previously published research. The investigation into the correlation between the concentration of filler and the strength of fractures is illustrated in Figure 3c,d. As the filler is incorporated, the fracture strength was observed to decrease. Due to the inadequate matrix-filler interaction between the matrix and Sm/La-PMN-PT particles, the mechanical properties deteriorate. Figure 3e,f depicted the relationship between the filler content of the composite and the frequency dependence of the dielectric constant and dielectric loss, respectively. This study revealed that the dielectric constant exhibited an upward trend as the concentration of Sm/La-PMN-PT particles increased, owing to the incorporation of inorganic fillers possessing a high dielectric constant [30,31,32]. In addition, the dielectric constant of Sm/La-PMN-PT/PVDF composites is higher than Sm/La-PMN-PT/PDMS composites because of the higher dielectric constant of the matrix itself. Meanwhile, the Sm/La-PMN-PT/PDMS composites show a much lower dielectric loss because the PDMS belongs to the non-polar elastomer. In addition, the similar spectra curves in both composites indicate that the introduction of the filler does not change the dielectric performance of the matrix and the properties of the composites are still dominated by the matrix [33,34].

**Figure 4:**
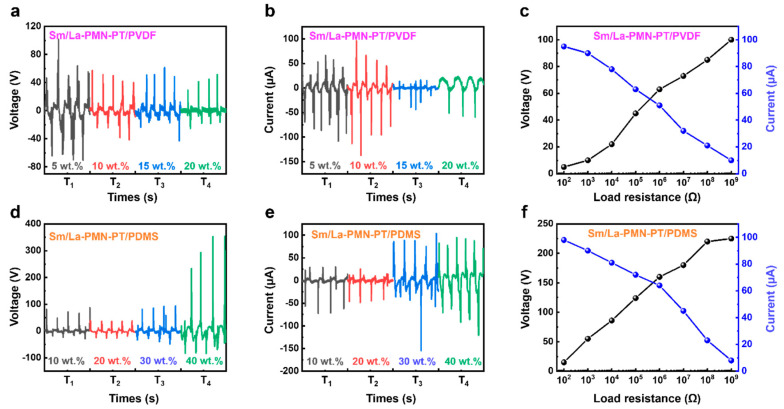
Output performance of PENG with different soft matrices. (**a**) The output voltage, (**b**) current, and (**c**) voltage/current with the external load resistance ranging from 10^2^ to 10^9^ Ω of 10wt.% Sm/La-PMN-PT/PVDF PENG device. The (**d**) output voltage, (**e**) current, and (**f**) voltage/current with the external load resistance ranging from 10^2^ to 10^9^ Ω of 40 wt.% Sm/La-PMN-PT/PDMS PENG device.

The electrical output performance of the fabricated Sm/La-PMN-PT/PVDF PENG and Sm/La-PMN-PT/PDMS PENG devices with varying concentrations is illustrated in Figure 4. In the same scenario (frequency, external force, etc.), the output voltage and current were measured. It was observed that the output voltage and current showed different trends: decreases with the content of ceramic filler in PVDF-based PENG, while increases with the content of ceramic filler in PDMS-based PENG. Figure 4a shows that 10 wt.% Sm/La-PMN-PT/PVDF generates the highest output, while 40 wt% Sm/La-PMN-PT/PDMS performs better, demonstrating concentration-dependent interactions between the filler and matrix. This can be attributed to the fact that the dielectric constant increased as the content of ceramic fillers increased. PDMS, as an elastomer, often exhibits substantially better mechanical properties and filler–matrix interactions. The greater capacity of PDMS-based composites to retain charge, resulting from a higher dielectric constant, can improve the efficacy of nanogenerators. However, the PVDF has observed that the mechanical and dielectric properties will alter substantially around the percolation threshold. That is why the composites can still generate a similar output voltage in Figure 4a, but the short circuit current reduces significantly with more than 10 wt.% filler, as shown in Figure 4b. The output voltage and current of PENG are 40 wt.% Sm/La-PMN-PT were nearly four times higher than those of PENG with 10 wt.%, as demonstrated. Figure 4d,e illustrates that the average output voltage and current of PENG with 40 wt.% were approximately 300 V and 100 μA, respectively. However, the PVDF-based composites may suffer decreased interaction between fillers and PVDF with the higher filler content. In addition, the output performance of the TENG device was examined in relation to external load resistance for practical applications, as illustrated in Figure 4c,f. The peak–peak voltage experienced a substantial increase as the external load increased from 10^2^–10^9^ Ω. Their output current values are equivalent. However, PDM-based composites exhibit a significantly higher output voltage due to the high content of rare-earth doped PMN-PT filler with high piezoelectric and dielectric properties. To better compare to pure PMN-PT and PZT filler, the output performance comparisons are listed in Table 1, in which some of the power data are calculated by the power density times device area. The comparison indicates that the types of piezoelectric filler strongly affect the output performance.

**Figure 5:**
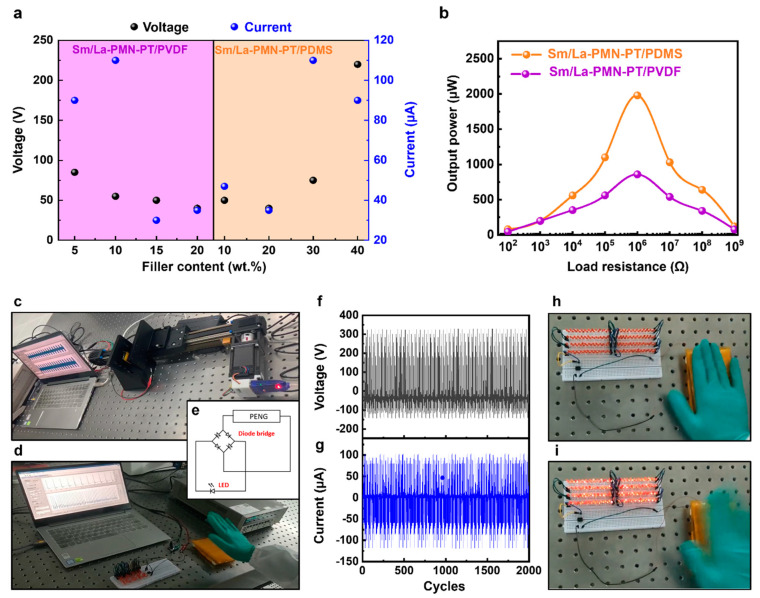
(**a**) The comparison of the output performance of Sm/La-PMN-PT/PVDF and Sm/La-PMN-PT/PDMS PENGs. (**b**) Output power of 40 wt.% Sm/La-PMN-PT/PDMS and 5wt.% Sm/La-PMN-PT/PVDF PENG devices with the external load resistance ranging from 10^2^ to 10^9^ Ω. (**c**) The photo of the measurement system for the durability test. (**d**) The photo of the measurement system for the LED lighting. (**e**) The schematic diagram of a diode bridge. The durability test of (**f**) voltage and (**g**) current of 40 wt.% Sm/La-PMN-PT/PDMS PENG. The rate is 1 cycle/s. (**h**,**i**) The photographs of commercial LEDs connected in series directly lightened by PENG during the pressing/releasing process.

Figure 5a shows the output power comparison of the output performance of Sm/La-PMN-PT/PVDF and Sm/La-PMN-PT/PDMS PENGs. It is clear that the Sm/La-PMN-PT/PDMS exhibited much higher voltage (220 V) and current (90 µA). The output power of 40 wt.% Sm/La-PMN-PT/PDMS and Sm/La-PMN-PT/PVDF devices with external load resistance ranging from 10^2^ to 10^9^ Ω are shown in Figure 5b. The peak output power of PENG is up to 2 mW, and it can be employed in many applications. Figure 5c,d depicts the PENG measurement equipment, which comprises an electrometer and a laptop. This system is used for conducting extended cycle testing and illuminating commercial light-emitting diodes (LEDs). An illustration of a complete rectifying diode bridge is provided in Figure 5e. The device can be linked to the load to convert alternating current (AC) into direct current (DC) for the purpose of charging energy storage devices, such as batteries or supercapacitors. In addition, as shown in Figure 5f,g, the output performance of voltage and current of the PENG is still stable after 2000 cycles. Photographic photos in Figure 5h,i demonstrate the instantaneous brightening of 200 LEDs by the power supplied from the PENG through the rectifier. This occurs when the force is released and when it is pressed, respectively. The findings suggest that the PDMS-based PENG device has the potential to serve as a self-sustaining power supply for wearable and portable electronic devices.

## 4. Conclusions

The Sm/La-PMN-PT/PVDF and Sm/La-PMN-PT/PDMS composite films were thoroughly examined and employed for energy harvesting purposes. The composites were produced using the spin-coating technique, with varying amounts of Sm/La-PMN-PT. Both the piezoelectric fillers and the matrix had a substantial influence on the output performance of the piezoelectric nanogenerator. The relationship between the dielectric constant and dielectric loss of composite films was examined in relation to both frequency and temperature. This study revealed a notable increase in the dielectric constant and loss as the concentration of nanoparticles increased, whereas it decreased with the frequency. The dielectric loss of the 40 wt.% Sm/La-PMN-PT. remained at a low level (<0.1). Sm/La-PMN-PT/PVDF exhibited high voltage (220 V) and current (90 µA), which is related to 2 mW of the peak output power. The PENG’s consistent and high electrical output power was employed to illuminate commercial LEDs, indicating that the device has the potential for energy harvesting in biomedical applications.

## Figures and Tables

**Table 1:** Comparison of output performance of flexible PENG devices.

Piezoelectric Filler	Matrix	V_max_ (V)	I_max_ (μA)	Power (μW)	Ref.
BaTiO_3_	P(VDF-TrFE)	45	2	9.12	[13]
BaTiO_3_	PDMS	200	0.24	160	[35]
PZT	PVDF	62.0	-	136.9	[36]
PZT	PDMS	152	17.5	1100	[37]
PMN-PT	PVDF	10.3	0.046	-	[22]
PMN-PT	P(VDF-TrFE)	8.5	3	45.7	[38]
PMN-PT	PVDF	0.12	-	10.59	[39]
Sm-PMN-PT	PDMS	0.53	1.08	6.62	[40]
PMN-PT	PDMS	70	20	105	This work
Sm/La-PMN-PT	PDMS	220	90	2000	This work

## Data Availability

The original contributions presented in the study are included in the article, further inquiries can be directed to the corresponding author.
